# 1024. Case Study. Reducing Intraoperative Hypothermia in Burn Surgery Patients

**DOI:** 10.1093/jbcr/irag033.473

**Published:** 2026-04-06

**Authors:** Kim Priban

**Affiliations:** Akron Children's Hospital, Hudson, OH

## Abstract

**Patient Presentation (age range, injury details, relevant history):**

Patient population for this quality improvement project were all patients undergoing operative procedures in the operating room for burn injuries.

**Clinical Challenges:**

Intraoperative hypothermia, defined as core temperature below 36.0°C, is a common and preventable complication that can contribute to coagulopathy, increased blood loss, delayed wound healing, and prolonged hospital stays. Burn patients are particularly vulnerable due to impaired thermoregulation and significant evaporative heat loss from open wounds. A review of operative cases in 2024 identified hypothermia in more than one-quarter of burn surgeries, highlighting the need for targeted interventions.

**Management Approach:**

Planned interventions include implementation of an endotracheal warming device, consistent use of warming blankets and warmed intravenous fluids, adherence to standardized room-warming protocols, and anesthesia staff training. Outcome measures will be obtained from prospective temperature monitoring documented at baseline, pre-induction, intraoperative, and post-operative phases. Hypothermia incidence will be tracked in real time, and changes will be assessed through run charts and iterative plan–do–study–act cycles.

**Outcomes:**

Baseline review of 114 operative cases demonstrated 26.3% with documented intraoperative hypothermia. Observed gaps included inconsistent adherence to warming protocols, variability in equipment availability, and lack of staff awareness regarding hypothermia incidence. Currently analyzing risk variations (TBA, age, comorbidities, BMI) to identify trends. Early intervention testing has begun, and complete data, including post-implementation outcomes, will be presented.

**Lessons Learned:**

Pre and intra-operative teams are often not aware of the importance of temperature regulation in burn patients, even less severe injuries. Findings showed that patients who had shorter duration cases and were older aged had less interventions taken and therefore had higher rates of hypothermia. Education of the nursing staff in all areas that touch burn patients are vital.

**Applicability to Practice:**

Hypothermia is a significant yet modifiable risk factor in burn surgery. Implementation of standardized warming practices and introduction of temperature-regulating interventions such as endotracheal warming, show promise in reducing hypothermia incidence and improving patient outcomes.

